# Textural Anatomy, or Histology Applied to Physiology and Pathology

**Published:** 1847-01

**Authors:** 


### Art. VIII.

*Anatomie de Texture, ou Histologie appliquée à la Physiologie et à la Pathologie.* Par Ad. Burggraeve, Professeur à la Faculté de Médecine de l'Université de Gand, &c. Deuxième édition.—*Gand.* 1845.

*Textural Anatomy, or Histology applied to Physiology and Pathology.* By Ad. Burggraeve, Professor at Gand.—*Gand,* 1845. 8vo, pp. 700.

M. Burggraeve's work professes to be an exhibition of the present state of microscopic anatomy, and of its application to pathology. So wide a subject cannot be treated with great minuteness in a single volume, not closely printed, of seven hundred pages. The abundance of material in so small a compass must involve the necessity of a representation of *conclusions,* and the omission of the *grounds* from which they are derived. Hence, in the outset, an appearance of shallowness, from the state of the case: the conclusions of science are often simple, when the process by which they have been reached is most elaborate. A physiologist is under especial disadvantage in this respect; so many of his conclusions are mere facts, that, if he is confined to their enumeration, he will differ but little, externally, from those authors whose office it is to write for the million.

Invaluable as physiology is, simply as a science, one of its most important bearings is, obviously, on human pathology. The *symmetry of disease* is the fact which gives its value to textural anatomy, and the *exactness* of that symmetry constitutes the difficulty of its application. It is this symmetry which has recommended the study of the eye, physiologically and pathologically, as exhibiting lesions plainly visible, which must find their counterparts in other parts unseen.

Histology is a science, including at once fluids and solids, and, in its pathological application, realizes in M. Burggraeve's view the anticipation of the old physicians, and unites a rational humoralism with a modified solidism.*

M. Burggraeve carries us through the formation of the primary cell, its development into tissues, and *their* union into organs. There is little or no novelty in this, the introductory, part of his work, and so much doubt has of late been thrown on the nature of the evolution of cell-life, that our system, as at present recognized, may have to undergo an entire remodelling, and our part may still be merely to collect facts for some future and more successful theory.

Before proceeding to our selection from M. Burggraeve's work of such parts as may represent its character, a few words may be spent on the principle which professes to be the basis of the histological theory. It is the enunciation of Carus, that, granting the original development of every organized body from a primary type of formation, "every superior degree of evolution of an organized structure consists in the multiplication of this type, constantly repeated at different and gradually increasing powers." This is a mathematical statement of a physiological fact, and it is dangerous ground when we apply the terms of one science to another, particularly when the sciences are so dissimilar as the two here concerned.† Luckily, experiment is always at hand to correct the fallacies into which this axiom, standing by itself, might lead; such, for instance, as the doctrine, that in the grades of organized beings there is one uniform line of development from the lowest to the highest, a doctrine simply opposed to the fact that many animals in an inferior order are higher in organic development than the lower animals in one superior: such as, again, the notion that one species is capable of development into a higher species, in confutation of which our appeal lies to the analogy of nature, and which, as connected with the introductory part of M. Burggraeve's work, merits a few moments' consideration.

There is, indeed, no antecedent reason for supposing that a simple cell should be developed in one form more than another: the circumstances in which it is placed determine the direction of its development. It follows from this, that, in the lowest instances of organization in vegetables, the fungous germs, according to surrounding circumstances, may exhibit different genera in their perfect state. To say this, seems to be admitting a great deal; but all "circumstances" are not "separable accidents," and the *parentage of the cell* is a circumstance which *determines* the character

* The inclusion of *fluids* in histology renders the name somewhat inappropriate; but names get recognized before substitutes are found, and it is often no easy matter for an objector to propose a better substitute. Has the old technicality of Anaxagoras any recommendations? We are in no danger of applying it as *he* did: καὶ τὰς μὲν "ὁμοιομερείας" (ἀπεφήνατο) ὕλην, τὸ δὲ ποιοῦν αἴτιον τὸν νοῦν τὸν πάντα διαταξάμενον. Pseudo-Plutarch. de Placit. Phil., lib. i, p. 876, D. Vid. Lucret., i, 830. *Homœopathy* is too good a word to be wasted on empiricism; it might be used in pathology to express the *symmetry of disease*, while *homœomery* would express its analogue in physiology.

† Butler, Introduction of Analogy of Religion to the Constitution and Course of Nature. When will another Butler arise to apply the newly discovered facts of physiology to this, their highest end.

of its evolution. It is in those cases where fecundation is most obscure that this capacity of various development exists: in the highest organization it does not exist at all; and between the two extremes the capacity may vary inversely with the rank held by the parent in the organized world. From one plant a hybrid may be produced by applying the pollen of a different species to its ovarium; from one animal a hybrid may be produced by the fecundating influence of an individual of a different species, but in neither case can the stock be permanently continued. When we come to man, hybridity ceases to be possible: the ovum is potentially a *human* being from the circumstance of its parentage; it is capable of development, but only in one direction and from one source. In no case can a hybrid *species* be perpetuated: in the highest, the human organization, a hybrid *individual* is impossible. Pasiphae's offspring was not an idea derived from physiology, nor yet the marvel told us by Herodotus.* Indeed, our author does not use this axiom of Carus, and we may complain of an *affectation* of deep views, when a high-sounding apophthegm is propounded as the basement of a theory, without rhyme or reason.

We have now to accompany our author in the treatment of his subject. And in few words his scheme may be stated as follows: given the cell-germ, to trace its development into a cell; to trace the subsequent changes of the cell, in the production of blood, &c., of fibre, nerve, and vessel, as well as in the production of abnormal growths; to follow up these secondary formations into systems, whether cellular, nervous, vascular, parenchymatous, locomotive, tegumentary; to mark the functions of these systems and their lesions, the preservation of the one or the reparation of the other; and to define how art can lend its aid to the restorative energy of Nature. Such is the task undertaken by M. Burggraeve, and the *plan* is unquestionably good; it is very much what a system of histology ought to be. The manner in which M. Burggraeve has accomplished his task is of course another question, and our quotations from his work will supply the data for the judgment to be passed on its merits.

We will begin by letting our author speak for himself; we shall best represent him by almost literal extracts; and while this is our object, we shall neglect the correction or indication of errors, which will easily be detected by our readers. After this representation of M. Burggraeve's powers and opinions we will confine ourselves to such statements as may seem novel or worthy of consideration.

The cell, says our author, persists in its original state in the fluids, the blood-globules, chyle-globules, and lymph-globules; in the first, the red colouring matter fixes the oxygen of the inspired air. On the *surface* of solids, the cell exists modified by external causes in the epidermis; by both internal and external in the various forms of epithelium; in the *substance* of solids we have corpuscles of gray nervous matter, of fat (frequently with crystals of cholestearine interspersed), of pigment (which in their pathological development give rise to schirrous, fungous, and other tumours), of bone, of cartilage, and of glands, whether tubular (in various degrees of development) or aciniform. With respect to the glands, M. Burggraeve insists upon their centripetal development; they are not

* Herodot., vii, 57, ἵππος ἔτεκε λαγόν.

prolongations of mucous membrane in the first instance, but separate formations, and afterwards united with the mucous system. That such is the case with the nervous medullary fibre, is established by M. Longet at the beginning of his invaluable work, and M. Burggraeve asserts it as a law of formation without exception.

*Fibres.* The development of fibre is with M. Burggraeve that which is ordinarily given, viz. through the elongation and terminal fission of nucleated cells. It is known that Mr. Paget would regard it rather as produced "from a structureless or dimly granular substance, first marked and then broken up into fibres." However this may be, the cavity of the fibre is commonly filled with a deposit which may consist either of earthy salts or of proteiniform compounds; the former class will contain: 1. The yellow elastic fibre, which forms the middle or proper coat of the larger arteries, the yellow vertebral ligaments, &c. The ashes of this substance, when burnt, consist mainly of phosphate of lime; and hence the apparent ossification of arteries in old age, which results, in fact, from an increase in the quantity of this deposit. This fibre occurs in two forms: the one scroll-like and interlacing, the other reticulated, as in the yellow ligaments between the laminæ of the vertebræ. 2. The eburnated fibres of the teeth, which, says M. Burggraeve, differ from the former only in the diminished quantity of water and animal matter which they contain; *both* species are wavy in direction, and frequently bifurcate at their extremities. The latter class contains the cellular, sclerous or tendinous, and muscular fibres, as well as the dartoid, which, by containing fibrine no less than gelatine, form the transitional state from the first two to the last. We shall have occasion hereafter to speak again of the dartoid fibre, which occupies a prominent place in M. Burggraeve's Histology. His view of muscular fibre (in which he agrees with Dr. Mandl, p. 561) differs from that generally entertained in England; the primitive fasciculus of fibrillæ, he says, owes its transversely striated appearance to a spiral filament winding round it.

*Nerves and vessels* close the list of secondary formations; both are perforated, and perhaps both are lined with a ciliated epithelium (here we can hardly restrain our pen from adding a note of admiration); so far they are analogous, but they differ in their contents, which are, in the nerve, a secondary deposit in the form of a pulp; in the vessel a fluid in constant motion. The nervous pulp imparts its white colour to the containing cellulo-elastic sheath, which is composed of a double fibre, revolving in inverse spirals; (have M. Burggraeve and Dr. Martin Barry never met?)

*Vessels* in animals have their analogues in plants. It was Boerhaave who defined an animal to be a plant turned inside out (indeed, in the fœtal state, the placental nourishment brings into still closer apposition the animal and vegetable kingdoms), the spongioles of a plant are the same with the lymphatic system in the animal; both end in blind extremities, and by them absorb their nourishment. M. Burggraeve maintains that in a villus the lymphatics embrace the terminal artery and vein, and that, in consequence, these two last do *not* reach the surface of the mucous membrane. (Be it remembered that we are representing M. Burggraeve, and withholding any critical protestations.) The lymphatics are formed by the union of contiguous cells, blood-vessels by prolongations sent out from the surfaces of primitive cells, and forming networks with greater or less interstices.

We may now, without fear of injustice to our author, cease to analyze, and adopt a more summary mode of proceeding with the rest of his work.

*The nervous system.* "Nerves," says M. Burggraeve, "bear the same relation to the solids of the body as the blood does to the fluids; they radiate like an atmosphere, the source of motion, sensation, and irritability." It is open to any one to propound a theory on the real constitution of the nervous system. The fact which is to be the keystone of the whole has not yet been discovered, or if it shall eventually prove to be a common one, like the fall of an apple, physiology has not yet had its Newton. M. Burggraeve has his theory; with him there are medullary fibres which form closed circles in the brain and spinal cord, and of them, the central part, the cerebro-spinal axis, consists: the peripheral part is represented by nervous cords, again forming continuous circuits, dipping centripetally into the gray matter of the cerebro-spinal mass. This hypothesis seems to our author more likely than any other to throw light on the nervous apparatus: it is not very apparent how it will tell so advantageously.

It is the opinion of M. Burggraeve (as it was of many old philosophers*) that sensation takes its birth properly in the tissues of the extremities. Here are found gray globules in the meshes of the nervous plexus and in the track of nerves of sensation; and sensation is conveyed by the nervous cords to the nervous centres for its development and its regular diffusion throughout the economy.

"These centres are to the dissemination of the nervous principle what the heart is to the distribution of the blood. The heart is the regulator not the principle of the circulation; the principle must be sought for in the capillary network of the extremities; even so, the organs are far from being passive media, traversed merely by the nervous currents; each of them is gifted with a certain power and spontaneity, whereby the emission of the nervous principle takes place constantly, in proportion to its needs. . . . . . . The terminal loops or plexuses correspond to the capillary network of blood-vessels. In organs of small extent, but requiring a large supply of nerves, as on the surface of the tongue, the papillary extremities of the nerves are formed into convoluted knots, presenting the greatest possible number of points of contact in the smallest possible space, representing in miniature the convolutions of the brain."

"Nowhere," says M. Burggraeve, "are the nervous cords continuous with the fibres of the central mass; the direction of the one class is for the most part contrary to that of the other." M. Burggraeve regards the central termination as *always* in loops, though he admits the opinion to be merely hypothetical. The researches of Kölliker have led to a modification of this theory. (See Paget's Report for 1844-5, p. 46.)

M. Burggraeve enters on the question whether the nerves of general sensation can supply the place of those of special sense. The question has been

* It *may* be, that they would have been of the contrary opinion had their acquaintance with anatomy been greater. For an instance, by way of illustrating our assertion, see Plotin., Ennead. iv, 4, c. 23, τὸ δὲ ὄργανον δεῖ ἢ πᾶν τὸ σῶμα ἢ μέρος τι πρὸς ἔργον τι ἀφωρισμένον εἶναι, οἷον ἐπὶ ἁφῆς καὶ ὄψεως.

handled by M. Longet* with his usual skill; and by him the question is decided in the negative. We do not think that M. Longet has received the attention from M. Burggraeve which he deserves; his name is *once* mentioned, just enough to show that his labours were not unknown (as indeed, how could they be?) to our author. M. Burggraeve ascribes the speciality of sense not to the essential character of the nerve, but to the modifying circumstances in which it is placed; and he meets the argument adduced by M. Longet, and derived from the fact that the irritation of one nerve of special sense never gives rise to the sensations proper to another, by observing, that the special character of the sensation was determined, in the first instance, by the *apparatus* in which the nerve was disposed (as the optic nerve, "in the most perfect and exact of dioptrical instruments"); and, that the nerve *retains* its *own* faculty of special sensation, thus stamped on it by habituation, in the same way as, by means of those nerves whose fibres had been distributed to an amputated limb, the sensation of pain is referred to the limb which has been lost. In some cases, a nerve of special sense is replaced by one of general sensation; for, in some animals, "the optic nerve, absent or atrophied, is supplied by the naso-ciliary branch of the ophthalmic of Willis." M. Burggraeve leaves undetermined how far the insertion of the nerve in the encephalon may contribute to the nature of the sensation, but remarks, that "if the corpora quadrigemina are the organs of luminous perceptions, the impressions which give rise to these perceptions may reach that part of the brain by *another* route than that of the optic nerve, as, by the trigeminus or the nerves of the medulla oblongata." How far M. Burggraeve would extend this power of substitution he does not distinctly affirm, but he throws in a hint that certain conditions "of magnetic somnambulism present equally striking cases of these singular transpositions." Generalization is a seductive thing; we do not *deny* that general sensation and special sense may be ultimately alike, but the argument which would reduce the nerves of special sense to the category of general sensation, modified by the organ, e. g. by a dioptrical instrument, would at least require the presence of a similar instrument for the conversion of a nerve of general sensation into one of special sense.

*The vascular system,* which takes its character in the higher animals from the centralization of respiration consists of the heart, the arteries, the capillaries, veins, and lymphatics. Its development is centripetal, and the heart is last in order of formation. The *capillaries,* the terminations of the vascular system, are—1, in the form of a *network,* where the tissues are to be nourished; 2, of a *plexus,* where they serve for diverticula, as in the pia mater, or for a protection against the effects of stagnation, as in the erectile tissues; and (3) of *villi,* where absorption is carried on, as in the intestines of the placenta.

The predominance of *veins* and *lymphatics* varies inversely; absorption by the former is lateral, by the latter terminal. This inverse proportion is particularly evident in the small number of lymphatics in the brain and pituitary membrane. The predominance of veins or lymphatics determines the bilious or the lymphatic temperament in different persons, or

* Tom. ii, p. 184.

of the same person at different tlmes. In excessive excitement of the venous system, the lymphatic absorption is arrested, and the activity of the venous is increased; hence the fearful effects of puerperal peritonitis, resulting from the intensity of venous absorption, and the remedial effect of venesection, by restoring the lymphatics to the exercise of their functions.

Experiments have proved to M. Burggraeve with respect to the *absorption* of gas, that the air found in the stomach of a horse, and analysed immediately after death, had lost 20 per cent. of oxygen and 15 per cent. of nitrogen, but that the carbonic acid had increased in the proportion of 32 per cent. There is, proportionally, more of the oxygen of the air introduced into the stomach absorbed, than of the air inspired by the lungs.

*Lymphatic ganglia.* In abscesses formed in these ganglia their structure is not lost; their vessels are spread over the walls of the cellular cyst and easily return, on the evacuation of the abscess, to their normal state.

*Lymphatics.* The external coat of the lymphatics is dartoid, and is found muscular in the horse; still further, lymphatic hearts are developed in the course of the lymphatics in the inferior vertebrata, (reptiles), and, we may add, in birds, from the researches of Professor Stannius.

The Pacchionian corpuscles are little clusters ascertained to consist of the densest possible network of blood-vessels and lymphatics; they are, in fact, as their discoverer imagined, lymphatic glands. A similar arrangement is demonstrated by M. Fohmann in the choroid plexus. That the Malpighian bodies in the spleen also belong to the lymphatic system is rendered probable from the relation which they bear in number and size to the lymphatic vessels (which vessels, moreover, can, in all appearance, be traced to them), and from the increase in their size during the period of excitement of the whole abdominal lymphatic system, namely, during digestion. M. Burggraeve entertains the same opinion of the Malpighian bodies in the cortical substance of the kidney;* "they are hollowed," says our author, "and give rise immediately to lymphatics."

*Diverticula.* M. Burggraeve has some observations, which we will gather from various parts of his work under this head, as worthy of consideration. 1. The *brain* is protected from the rupture of its delicate vessels, not only by the course of the vertebral and carotid arteries, and the unyielding texture of its sinuses, as well as by the minute caliber of its capillaries, which requires an elongation of the red corpuscles for their transit, but by the existence of *diverticula.* These are the choroid plexuses, which receive the "bloody lymph," till they can be carried off by the veins. 2. In the *eye,* the choroid coat with its vasa vorticosa surrounds the nervous substance, as the pia mater surrounds the brain, and, like the choroid plexuses of the latter, the *ciliary processes,* are a *diverticulum* by which the sudden alternations of an erectile tissue like the iris, are prevented from being hurtful. This curious analogy of the eye to the brain may be extended by observing that the veins of the iris pour their contents into a sinus before emptying themselves into the ophthalmic veins, while these again pour *their* contents into the cavernous sinus: and, moreover, the

* So does Professor Hyrtl. See Paget's Report, 1844-5, p. 40. Mr. Paget rejects the opinion.

capillaries of the retina fall far short in diameter (as in the brain) of the blood-corpuscles, which are consequently retarded and obliged to elongate themselves. 3. In efforts, the return of the blood by the internal jugular vein is impeded; here again the brain is protected from venous congestion (not only by the sinuses) but by the *diverticulum* which the blood finds in the *thyroid body;* and the same protection is supplied during the less violent but more continuous effects of speaking. The veins of the thyroid body have, as might be anticipated, no valves. 4. The *spleen* is to the abdominal viscera what the thyroid body is to the cerebral; engorgement of the portal system is prevented by this office of the spleen in man, as the embarrassment of circulation is obviated in diving animals by a dilatation of the vena cava inferior. The pain felt in the left hypochondrium in running (*à fortiori*, after a full meal) is due to an excessive demand on the spleen as a diverticulum. It was this *pain* which led the ancients to the use of medicaments with a view to the extirpation of the spleen in foot-racers, a proceeding by no means in accordance with physiology.* 5. The *great omentum* too serves as a *diverticulum* to receive the superfluous blood of the stomach, in the intervals of digestion. And, lastly, 6. The *liver* has for one of *its* functions, that of a *diverticulum;* it varies in size inversely with the spleen, the extirpation of the latter being followed by a considerable enlargement of the former, and a pain in the *right* hypochondrium may be produced analogous to the pain above mentioned in the left.

After the vascular system, M. Burggraeve proceeds to the *parenchymatous*. We have already anticipated some of his observations, as we have preferred collecting those together which bore on one point, to following the exact order of his work. He divides the parenchymata into—1, the sanguineous ganglia, viz. the spleen, the thyroid body, the thymus, the supra-renal capsules; 2, the lymphatic ganglia; 3, the branchial and bronchial apparatus, viz. the branchia of the fœtus, the placenta, the lungs, and the liver; 4, the glands proper, viz. for secretion, digestion, reproduction, lubrifaction.

Our convenience leads us to introduce here the observations made by M. Burggraeve in various parts of his work on the *dartoid tissue*. This seems to be the same with the "contractile fibro-cellular, or connective, tissue" of Henle and others. The dartoid fibres belong to the fibrous class of secondary formations, with proteiniform deposit, and are intermediate between cellular and muscular fibres, When irritated, they have a tendency to contract, as is seen in the cutis anserina and in the effect of cold upon the scrotum; if stretched, they return to their normal length on the removal of the physical extension.

As fibrous tissue with *earthy* deposit forms the proper and middle arterial coat, so the dartoid or fibrous tissue with proteiniform deposit forms the external, and by its toughness continues entire after the laceration of the proper coat (as by ligature). Dartoid tissue forms the external coat of the *veins*, and becomes muscular in the pericardiac portion of the pulmonary veins and in the venæ cavæ of large animals (as in the ox): the external coat of the lymphatics is dartoid also, but of greater strength;

* Vid. Plin., Hist. Nat., xi, 80; xxvi, 83.

the skin is in different parts extensible, elastic, or contractile, in proportion to the predominance of cellular, elastic, or dartoid tissue in the derma. In the parenchymata the dartoid tissue plays an important part. The spleen is invested by a dartoid coat, which, at the hilus, is prolonged into the organ, inclosing its vessels, and following them in their ramifications, and the framework is completed by laminæ and threads of the same tissue proceeding from the inner surface of the coat and uniting with those which accompany the vessels. The distensibility of the tissue admits of an increased flow of blood into the organ, and its contractility restores the organ to its natural size, when its office as a diverticulum is no longer required. The kidneys are supplied with the same bridles; and in the liver they proceed from Glisson's capsule* to the proper fibro-cellular coat of the organ. This arrangement provides in a striking way against any pressure on the portal circulation, for the canal in which the portal vein and hepatic artery run is dilated rather than otherwise, while the dartoid filaments, by their contraction, diminish the bulk of the liver. On the other hand, the hepatic vein is channeled out in the parenchyma of the organ, and the contraction of its parietes can, says M. Burggraeve, only promote the centripetal circulation.

M. Burggraeve, who has not a few new notions on subjects generally agreed to, has of course one upon the spleen. We have already quoted his account of it as a diverticulum. With respect to the splenic juice or matter (*boue de la rate*), we learn, says M. Burggraeve, that the spleen is developed in inverse ratio to the liver, and that this splenic matter varies directly with the quantity of blood-corpuscles.† It consists of the detritus of the blood-corpuscles, of some of these corpuscles themselves *diminished in size*, of much albumen, of a little fibrine, and of various salts. These are submitted to the absorbents for redintegration into blood, and by their separation also (which is not, however, a *necessary* process), the blood is prepared to undergo the action of the liver, and the due proportion of red globules in the blood is preserved.

M. Burggraeve insists on the resemblance in structure and function between the liver and the lungs: the terminal vessels are arranged in the former precisely as in the latter, except that the terminal cell of the hepatic duct is interposed between the capillaries of the liver instead of the terminal air-vesicle. It will be seen that he differs in this from Mr. Kiernan, and he attempts to account for (as he conceives) Mr. Kiernan's error. It will require strong evidence for us to relinquish the results of our countryman's sagacity. The different theories on the structure of the liver and the lungs are in singular contrast. From the analogy of birds, in which the terminations of the bronchi are not closed sacs, but form a labyrinthine network, opening on the pleural surface, into large air-receptacles, which lie in the intervals of the viscera, and even of the bones. M. Bourgery makes the extremities of the bronchi in man and in mammalia communicate

* M. Burggraeve wishes to call this capsule by the name of his countryman, Van de Waele, as having preceded Glisson in describing it. It may be so; and, in that case, of course Glisson has no right to rob Van de Waele of his credit. But we doubt whether any individual's name should be foisted into the nomenclature of science. Witness the absurdities of our botanical nomenclature.

† So Pliny says, Lienem non esse constat iis quæ careant sanguine, xi, 80; and Aristotle, affirmatively, σπλῆνα δὲ τὰ πλεῖστα ἔχει ὅσαπερ καὶ αἷμα, Hist. Anim., ii, 15.

in capillary canals. As M. Bourgery thus assimilates the pulmonary structure to the hepatic (as we regard the latter in England), so M. Burggraeve reduces the hepatic to the recognized view of the pulmonary.

From the parenchymatous structures, M. Burggraeve proceeds to the fluids, and we find nothing which calls for special notice in his observations on them. We pass on then to the tissues connected with motion, and here, too, we have little or nothing to remark. He divides them into passive, as bones, cartilages, &c., and the active, which he distinguishes into—1, the vibratile cilia; 2, the cavernous or hæmomyary; and 3, the muscular or neuromyary. M. Burggraeve is not successful in his nomenclature: "*αἶμα, sang,*" and "*μύειν, mouvoir,*" will hardly reconcile us to such hard substitutes as the above for "erectile" and "muscular." Can it be that our author thinks he has discovered the root of the word "muscle" itself, and that the resemblance in form, which is supposed to have given occasion to the name, does not satisfy him? Whether this be so or not, it is most certain that our author, when he comes to Greek, *μύων βαδίζει.**

The division of muscles into those of animal and those of organic life, does not satisfy M. Burggraeve, as being an *imperfect* division; and he complains equally of the vagueness of the terms voluntary, involuntary, and semi-voluntary: he suggests, therefore, the division into "muscles with levers," and "muscles without levers;" but how this division is preferable to the others does not appear, since, manifestly, the latter class will place in the same category the visceral, vermicular muscles, and the *Platysma myoides* and *Palmaris brevis.* The fact is, few things are effected in nature *per saltum,* and where we find vagueness (if so be) in things, we must make allowance for vagueness in words.

From M. Burggraeve's account of the tegumentary system, it is only necessary to refer to his observations on the mucous membrane of the tongue. It is well known that the papillæ of this organ have recently been examined by Dr. Todd and Mr. Bowman. M. Burggraeve differs from them and from M. Cruveilhier in his opinion concerning the *Circumvallatæ papillæ.*

"Frequently," he says, "the mucous membrane lining a follicle is elevated by subjacent glandular corpuscules. This gives the appearance of a papilla standing up from a depression, and surrounded by a raised margin; by this means the name of papillæ has been applied to bodies, wholly unentitled to the name, as in the case of the lenticular papillæ distinguished by M. Cruveilhier under the denomination of perforated or calyciform. It is plain that neither their structure nor their function justifies us in identifying them with nervous papillæ: they are glands."

We conclude our review as we began it: M. Burggraeve has essayed a wide task in a small compass: and his space has been still more curtailed by mere catalogues of the facts of topographical anatomy. Such are his enumerations of the muscles, aponeuroses, blood-vessels, and nerves. If in some parts our extracts have been few, our readers are at liberty to infer that, as it has not been our object to epitomise the work, but to abstract from it views which lay claim to novelty, the portions thus lightly touched on contain a rapid but faithful conspectus of the con-

* We may take occasion here, too, to protest against the unscholarlike *look* of Greek words written without their proper accents.

clusions of human physiology up to the time of the composition of the work. We add this qualification because, though we have not the first edition, we have evidence which leads us to suspect that it has not been so *improved* in the second edition, as to keep pace with the rapid evolution of physiological research. For instance, M. Burggraeve ascribes to Dumas an opinion which he is well known to have revoked: analyses, again, of certain fluids given by M. Burggraeve have undergone considerable alteration, and theories which he admits have been laid aside. It may be said, that the multitude of modifications of opinion which each year produces may well excuse an author's ignorance of some of them, and we admit this to the full. We even think that the very endeavours to throw into a consistent whole the facts which are daily brought to light in physiology is laudable, and the more so, because, when physiology, now in its infancy, shall be a few years older, we may have to look back on our present position from a distance far in advance, and books which are now text-books will have become obsolete:

Tam venerabile erit præcedere quatuor annis.

